# Telemedicine Uptake During and After Pandemic-Era Deregulation in Japan

**DOI:** 10.1001/jamanetworkopen.2025.53150

**Published:** 2026-01-09

**Authors:** Kazuki Ohashi, Kazuhiro Abe, Yoko Shizawa, Zhao Jieyu, Machiko Ukai, Shigekazu Komoto, Katsuhiko Ogasawara

**Affiliations:** 1 Faculty of Health Sciences, Hokkaido University, Sapporo, Hokkaido, Japan; 2 Department of Health Care Policy, Faculty of Medicine, Hokkaido University, Sapporo, Hokkaido, Japan; 3 Institute for Integrated Innovations, Hokkaido University, Sapporo, Hokkaido, Japan; 4 Graduate School of Health Sciences, Hokkaido University, Sapporo, Hokkaido, Japan; 5 Department of Nutrition, Teine Keijinkai Hospital, Sapporo, Hokkaido, Japan; 6 Teine Family Medical Clinic, Sapporo Hokkaido, Japan; 7 Faculty of Engineering, Muroran Institute of Technology, Muroran, Hokkaido, Japan

## Abstract

This cross-sectional study assesses the uptake of telemedicine during and after the COVID-19 pandemic–era deregulation period in Japan.

## Introduction

The COVID-19 pandemic accelerated telemedicine use worldwide. In 2022, 39.3% of US adults reported using telemedicine at least once in the previous 12 months.^1^ In Japan, telemedicine was expanded to initial and follow-up visits under COVID-19–related deregulation starting in April 2020. An internet-based survey by Miyawaki et al^2^ found that only 4.7% of adults aged 18 to 79 years used telemedicine early in this deregulation. The deregulation ended on August 1, 2023, after which telemedicine was required to be video-based care at preregistered facilities.^3^ Although Japan’s telemedicine environment changed markedly after the pandemic, its post-COVID-19 period use and age-specific changes before and after the deregulation remain unclear. Evidence from claims data rather than self-reports has been lacking. This study describes telemedicine use across all ages using claims data covering the deregulation and postderegulation periods.

## Methods

We conducted a descriptive cross-sectional study using monthly aggregated outpatient claims data across all specialties from 2 public health insurance systems, covering approximately 35% of the total population in Hokkaido, Japan (eTables 1 and 2 in Supplement 1). Telemedicine use was identified using reimbursement codes (eTable 3 in Supplement 1). Age-specific proportions were calculated by 5-year age groups (eg, ages 0-4, 5-9, …, ≥100 years) throughout the study period (April 2022 to December 2024), and monthly trends were described by 5 age groups in the proportion of telemedicine use. This study was approved by the institutional review board of the Faculty of Health Sciences, Hokkaido University, which deemed the study exempt from informed consent because it involved nonhuman participant research and fully anonymized data. We followed the STROBE reporting guideline. Analyses were performed using R, version 4.3.3 (R Foundation for Statistical Computing).

## Results

**Figure 1.**
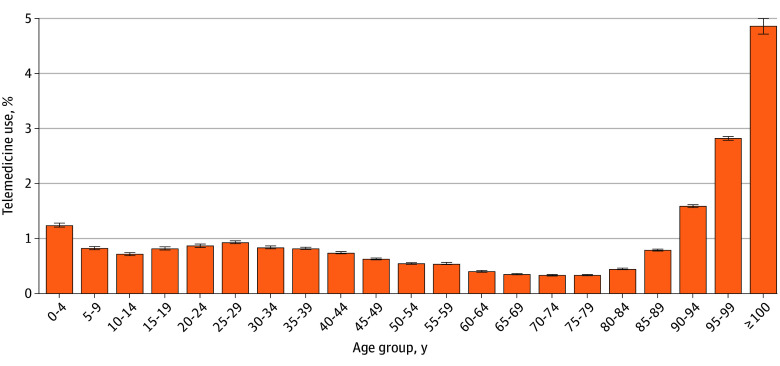
Proportion of Telemedicine Use by Age Group Outpatient visits conducted via telemedicine in Japan from April 2022 to December 2024. Error bars indicate 95% CIs.

**Figure 2.**
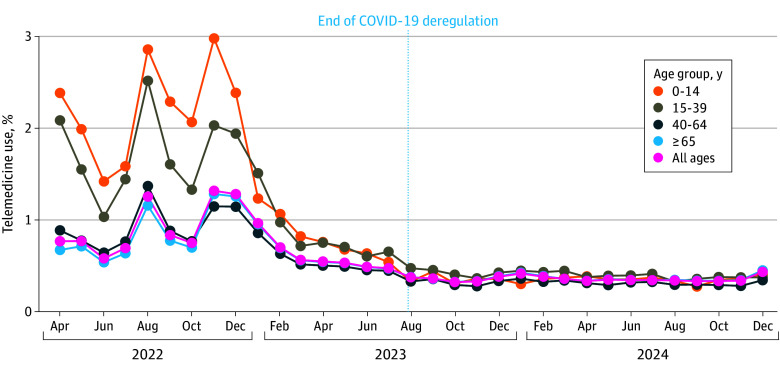
Trends in the Proportion of Telemedicine Use by Age Group Monthly proportions of outpatient visits conducted via telemedicine in Japan.

Among 77 040 681 outpatient visits recorded, 429 036 (0.6%) were via telemedicine, including telephone and video consultations. Telemedicine use exceeded 1.0% only among patients aged 0 to 4 years and older than 90 years, whereas all other age groups showed proportions below 1.0%, particularly less than 0.5% among patients aged 60 to 84 years (Figure 1). The monthly proportion of telemedicine fluctuated in 2022, mainly in the 0-to-14–year and 15-to-39–year age groups. Since March 2023, however, the proportion has remained below 1.0% across all age groups. The younger age groups (aged 0 to 14 years and 15 to 39 years), which showed relatively higher use in 2022, declined thereafter, staying below 0.5% after deregulation ended (Figure 2).

## Discussion

The findings of this cross-sectional study showed that telemedicine use remained below 1.0% of outpatient visits during the late-COVID-19 to post-COVID-19 period, far lower than in the US.^1^ Several factors may explain the limited adoption of telemedicine in Japan. Despite the need for investment in digital devices and secure communication systems, reimbursement for telemedicine remains lower than that for in-person visits,^4^ resulting in limited financial incentives for clinicians. Initial consultations are recommended to be conducted in person, and the prescription of narcotics and psychotropic agents is restricted.^3^ Telemedicine is encouraged only upon patient request,^3^ whereas in the US, 72.7% of users reported using telemedicine following a clinician’s recommendation.^1^ From the user’s perspective, following the end of deregulation, many patients appeared to return to conventional in-person care. Patients and clinicians perceive face-to-face consultations as safer and less burdensome than digital interactions.^5^ Although older adults prefer telephone consultations,^6^ video-based communication is recommended in Japan, which may pose an additional barrier for individuals with limited digital literacy. Relatively higher telemedicine use was observed among the oldest and youngest age groups, which may reflect selective use among homebound older adults or young children supported by family members. This study has a limitation of potential selection bias, as our findings may not represent those covered by employment-based health insurance. Nevertheless, telemedicine remains far from widespread in Japan, underscoring the need for stronger financial incentives for clinicians and better user support to promote its uptake.

## Notes

Supplement 1. **eTable 1.** Characteristics of the 2 Public Health Insurance Systems in Japan **eTable 2.** Characteristics of Hokkaido, Japan **eTable 3.** List of Reimbursement Codes for Telemedicine

Supplement 2.
Data Sharing Statement
