# Supplementary material for: Telemedicine Uptake During and After Pandemic-Era Deregulation in Japan
Source: JAMA Netw Open. 2026 Jan 9;9(1):e2553150. doi: 10.1001/jamanetworkopen.2025.53150 (PMC12789947; doi:10.1001/jamanetworkopen.2025.53150)
Supplement: Supplement 1. — eTable 1. Characteristics of the 2 Public Health Insurance Systems in Japan eTable 2. Characteristics of Hokkaido, Japan eTable 3. List of Reimbursement Codes for Telemedicine [file jamanetwopen-e2553150-s001.pdf]

Supplemental Online Content

Ohashi K, Abe K, Shizawa Y, et al. Telemedicine uptake during and after pandemic-era deregulation in Japan. *JAMA Netw Open*. 2026;9(1):e2553150. doi:10.1001/jamanetworkopen.2025.53150

eTable 1. Characteristics of the 2 Public Health Insurance Systems in Japan

eTable 2. Characteristics of Hokkaido, Japan

eTable 3. List of Reimbursement Codes for Telemedicine

This supplemental material has been provided by the authors to give readers additional information about their work.

© 2026 Ohashi K et al. *JAMA Network Open*.

eTable 1. Characteristics of the 2 Public Health Insurance Systems in Japan

	**National Health Insurance (NHI)**	**Late-Stage Elderly Medical Care System**
**Population**	Residents under 75 years of age who are not covered by employee-based insurance, including the self-employed, farmers, students, children, unemployed persons, and retirees	Adults ≥ 75 years and those aged 65–74 with certain disabilities
**No. insured persons**	1,082,922 (2022.4.1)*	861,637 (2022.9.30)*

* Statistics of Japan. e-stat. Available from https://www.e-stat.go.jp/en

© 2026 Ohashi K et al. *JAMA Network Open.*

eTable 2. Characteristics of Hokkaido, Japan

Item	Description
Geographic area*	Approximately 83,000km^2
Population*	5.22 million
65 years and over	1.66 million
75 years and over	0.85 million

* Statistics Bureau of Japan, Ministry of Internal Affairs and Communications, 2020 Population census.

© 2026 Ohashi K et al. *JAMA Network Open*.

eTable 3. List of Reimbursement Codes for Telemedicine

Category	Reimbursement code
Initial visit	111000110, 111011810, 111012510, 111012610, 11012710, 111012810
Follow-up visit	112007410, 112008350, 112011310, 112011710, 112015810, 112016310, 112016410, 112016550, 112016610, 112016850, 112017010, 112017610,
Initial online visit	111013850*, 111014210, 111014310, 111014410, 111014510, 111014610, 1111014710, 11016150*,
Follow-up online visit	112007950, 112008850, 112016750, 112016950, 112017150, 11203350, 11203450, 11203550, 11203650, 11203750, 11203850, 112024210, 112024710, 112024950, 112025050, 112025010, 112025150, 112025210, 112025310, 112025450, 112025650, 112025710, 112025850, 112025910, 112026010, 112026110, 112026750*, 11206850*,

*The pandemic-related special measures for telemedicine were terminated on July 31, 2023.

© 2026 Ohashi K et al. *JAMA Network Open.*
