# Supplementary material for: Telemedicine Uptake During and After Pandemic-Era Deregulation in Japan
Source: JAMA Netw Open. 2026 Jan 9;9(1):e2553150. doi: 10.1001/jamanetworkopen.2025.53150 (PMC12789947; doi:10.1001/jamanetworkopen.2025.53150)
Supplement: Supplement 2. — Data Sharing Statement [file jamanetwopen-e2553150-s002.pdf]

Data Sharing Statement

Ohashi. Telemedicine Uptake During and After Pandemic-Era Deregulation in Japan. *JAMA Netw Open*. Published January 09, 2026. doi:10.1001/jamanetworkopen.2025.53150

Data
Data available: No
